# The role of tumor-associated macrophages in HPV induced cervical cancer

**DOI:** 10.3389/fimmu.2025.1586806

**Published:** 2025-04-08

**Authors:** Zeping Chen, Binzhu Zhao

**Affiliations:** Department of Gynecology, Chengdu Pidu District Hospital of Traditional Chinese Medicine, Chengdu, China

**Keywords:** macrophage, cervical cancer, HPV, immune response, TAM recruitment, vaccine, diagnosis

## Abstract

Human papillomavirus (HPV), a double-stranded DNA virus linked to various malignancies, poses a significant global public health challenge. In cervical cancer, persistent infection with high-risk HPV genotypes, particularly HPV-16 and HPV-18, initiates immune evasion mechanisms within the tumor microenvironment. The polarization of tumor-associated macrophages (TAMs) from M1 to M2 phenotypes promotes cervical carcinogenesis, metastasis, and therapeutic resistance via establishing an immunosuppressive microenvironment. This review provides a comprehensive overview of HPV-induced immune evasion pathways, including MHC downregulation, T-cell impairment, regulatory T cell induction, and cGAS-STING pathway inhibition. Furthermore, describe the pivotal role of TAMs in cervical cancer progression, focusing on their phenotypic plasticity, pro-tumoral functions, and potential as therapeutic targets. By elucidating these cellular and molecular dynamics, this review aims to support advanced research. Targeting TAM polarization through immunotherapies and nanomedicine-based strategies represents a promising strategy for enhancing patient outcomes.

## 1 Introduction

Human papillomavirus (HPV) induces hyperproliferation of squamous epithelium in human skin and mucous membranes (1). High-risk types, notably HPV-16 and HPV-18, are strongly linked to malignant tumors such as cervical, anal, and oral cancers, collectively termed HPV-related tumors. While the immune system typically clears HPV in immunocompetent individuals, certain immune evasion strategies enable progression to malignancy. Recent studies have detailed HPV’s mechanisms to evade host adaptive immunity, closely associated with HPV-related tumor development.

Cervical cancer has seen significant advancements in prevention and diagnosis, including vaccines, HPV detection, cytological screening, and colposcopy. Persistent infection with high-risk HPV-16 and HPV-18 is the primary driver of cervical cancer. The tumor microenvironment (TME) (2–5), composed of tumor cells, bone marrow-derived cells, and stromal cells, fosters an immunosuppressive microenvironment that promotes tumor immune evasion, growth, and metastasis (6–8). Despite these insights, treatment outcomes for advanced and recurrent cervical cancer remain suboptimal. TAMs within the TME play a pivotal role in tumorigenesis and progression by promoting invasion, migration, angiogenesis, and suppressing anti-tumor immunity (9, 10). This review consolidates current knowledge on TAM polarization, their functional roles, and therapeutic targets in cervical cancer, aiming to advance research on its progression and treatment.

## 2 Mechanisms of HPV-induced evasion of host adaptive immune responses

### 2.1 MHC downregulation to prevent cytotoxic T lymphocyteactivation

CTLs are crucial in anti-tumor immunity, eliminating mutated or tumor cells through perforin-granzyme and Fas-FasL/TNF-TNFR pathways. Their ability to recognize exogenous peptides is governed by MHC class I and II molecules, with MHC contributing to variability in immune responses—a critical regulatory mechanism. Tumors frequently harbor mutations that disrupt MHC antigen presentation pathways (11). Genome-wide association studies have identified HA locus mutations potentially influencing susceptibility to HPV-related cancers. HPV employs gene expression modulation to inhibit antigen presentation, preventing viral antigens from being displayed on MHC class I molecules during infection. Specifically, the HPV E5 protein induces late endosome alkalization, disrupting the trafficking of peptide-MHC class I complexes to the cell surface and hindering the presentation of tumor-associated antigens, thereby suppressing CTL activation (12). In head and neck squamous cell carcinoma (HNSCC), HPV E5 overexpression confers CTL resistance by reducing antigen presentation, while treatment with the HPV E5 inhibitor gemcitabine can enhance MHC class I expression and mitigates this effect (13).

### 2.2 Regulation of CD4^+^ T cell activation

CD4^+^ T cells regulate CTL activation and secrete cytokines and chemokines that support anti-tumor immunity. In murine models, CD4^+^ T cell depletion impairs CTL responses to the HPV E7 protein (14). Clinically, HIV-infected patients with CD4^+^ deficiency exhibit a higher incidence of HPV-related cancers. In cervical cancer, CD4^+^ T cells exhibit impaired responses to HPV peptides, contributing to tumor immune tolerance (15). Anti-HPV activity is associated with CD4^+^ T cell phenotypes rather than quantity, with the CD4^+^CD161^+^ subset linked to improved survival (16, 17). In HPV-related oral cancers, CD4^+^ T cell abundance does not predict prognosis (18). Cervical cancer patients display a Th2-skewed CD4^+^ response and reduced IFN-γ levels (19). Conversely, in HPV-related oropharyngeal carcinoma, Th1 responses mediated by CD161^+^ and CD103^+^ T cells are prognostic (16). Furthermore, stromal fibroblasts secrete CCL20, driving pro-tumorigenic Th17 responses during invasive carcinoma progression, although their persistence across stages remains unclear (20).

### 2.3 Induction of regulatory T cell generation

Tregs suppress immune responses, disrupting normal host immunity. HPV-related tumors induce Tregs and other regulatory cells to inhibit anti-tumor immunity. Clinically, Treg levels are higher in HPV-infected individuals than in healthy controls, with chronic infections showing greater Treg elevations than those who clear the virus, suggesting impaired cellular immunity allows persistent infection (21, 22). Cervical HPV infections are typically localized to lesions with rare viremia, making local immunity crucial for infection outcomes. In cervical cancer, low-proliferative Foxp3^+^ Tregs are present at primary tumors, metastatic lymph nodes, and peripheral blood, indicating systemic immune tolerance and potential tumor metastasis (23). Studies on Treg levels in HPV-positive versus HPV-negative HNSCC are inconsistent (24, 25). However, the Treg/CD8^+^ T cell ratio remains a prognostic marker in HNSCC irrespective of HPV status (26).

### 2.4 Regulation of the cGAS-STING pathway

The cGAS-STING pathway senses exogenous DNA by synthesizing cGAMP from ATP and GTP, triggering STING, IRFs, and NF-κB to induce interferons and pro-inflammatory cytokines. While it mediates antiviral effects against DNA viruses, HPV evades this defense through mechanisms like HPV18 E7’s LCXCE domain blocking STING (27) and HPV16 E7 destabilizing STING via NLRX1 (28). Depletion of NLRX1 boosts type I interferon-dependent T cell infiltration and tumor suppression (29). In HPV16-positive HNSCC, the LCXCE domain of HPV16 E7 may disrupt cGAS-STING signaling (30). STING expression correlates positively with tumor-infiltrating lymphocytes and improved survival outcomes (31). HPV-positive HNSCC exhibits higher STING mRNA levels than HPV-negative cases, suggesting partial pathway activation despite HPV-mediated inhibition. STING activation may also enhance cetuximab-induced NK cell activity, driving tumor regression (32).

### 2.5 PD-1/PD-L1 immune checkpoint regulation

The PD-1/PD-L1 immune checkpoint is a conserved inhibitory mechanism regulating immune responses and plays a key role in inducing tumor immune tolerance during tumorigenesis (33–36). HPV-related tumors upregulate PD-1/PD-L1 to enhance immune tolerance. Preliminary analysis of 27 HNSCC tumors shows higher PD-L1 expression in HPV-positive versus HPV-negative tumors (30). In a study of 214 oropharyngeal cancer patients, 85.2% of HPV-positive cancers expressed PD-L1 compared to 57.1% of HPV-negative ones, and HPV-positive tumors also exhibited greater T cell infiltration (31). Additionally, IFN-γ secretion by T cells upregulates PD-L1 expression, potentially intensifying the inflammatory response in the tumor microenvironment. Analyses of TCGA and MSK-IMPACT cohorts indicate that HPV-positive status is a superior predictor of HNSCC outcomes compared to immune checkpoint inhibitor responses, independent of PD-L1 levels, and correlates with higher expression of inflammatory genes and CD8^+^ T cell infiltration in HPV-positive HNSCC tumors (37).

In summary, HPV-infected tumor cells evade host adaptive immunity through multiple mechanisms, including downregulating MHC molecules, suppressing CD4^+^ T cells, inducing regulatory T cells, and disrupting signaling pathways, thereby facilitating tumorigenesis. Establishing effective cellular immunity is essential for eliminating persistent HPV infections. Further investigation into HPV-related immune evasion will aid the development of vaccines, anti-tumor therapies, and prognostic tools.

## 3 The role of TAMs in cervical cancer

### 3.1 Phenotypic alterations of macrophages during cervical cancer progression

Macrophages exhibit a strong correlation with cervical intraepithelial neoplasia (CIN) progression, with their prevalence increasing linearly alongside tumor advancement (38). Human HPV manipulates innate and adaptive immune responses through various mechanisms, enabling immune evasion and persistent infection. In low-grade CIN (CIN I-II) associated with HPV, pro-inflammatory cytokines and infiltrating inflammatory cells are markedly reduced, fostering malignant transformation. While immune cell infiltration escalates from high-grade CIN (CIN III) to invasive cervical cancer, immune suppression endures, with the phenotype and function of infiltrating immune cells being tightly regulated.

Macrophage phenotypes dynamically shift across cervical cancer stages (39), contributing to tumor proliferation, invasion, and metastasis through multiple pathways. Within the cervical cancer microenvironment, monocyte differentiation into dendritic cells is impaired, while prostaglandin E2 (PGE2) and interleukin-6 (IL-6) secreted by cancer cells induce differentiation into M2 macrophages (40). Cervical cancer cells convert M1 to M2 macrophages, consistent with the high prevalence of M2 types in the tumor microenvironment (41). Supernatants from cancer cell lines stimulate macrophages to secrete elevated IL-6, IL-10, MCP-1, IL-8, GM-CSF, PDGF-AA, PDGF-BB, and VEGF. IL-6 and VEGF promote angiogenesis and tumor growth, while IL-4, MCP-1, and other cytokines create an immunosuppressive environment. GM-CSF and IL-6 synergistically enhance M2 polarization (42). Furthermore, cervical cancer cells reduce STAT1 and NF-κB p65 phosphorylation in M1 macrophages while increasing STAT6 phosphorylation to activate M2 macrophages, thereby impairing macrophage-mediated anti-tumor immunity.

### 3.2 TAM polarization facilitates HPV-induced cervical cancer progression

HPV comprises three genomic regions: six early genes (E1, E2, E4, E5, E6, E7), two late genes (L1, L2), and a non-coding region. Persistent infection with high-risk HPV types is the primary driver of cervical cancer. Studies demonstrates a positive correlation between cervical cancer progression and the expression of CD68^+^ or CD163^+^ macrophages, with macrophage infiltration increasing linearly with high-risk HPV infections (43). In HPV-positive cervical cancer specimens, M2-type macrophages are significantly enriched and exhibit elevated expression of antigen presentation genes, including CD74 and HLA-A (44). P-selectin glycoprotein ligand 1 (PSGL-1) in macrophages reprograms their function to activate T cells, contributing to anti-tumor immunity. In HPV16/18-positive CIN tissues, PSGL-1 expression is closely linked to infection status, lesion severity, immune infiltration, and prognosis (45). The HPV E6/E7 genes suppress CCL20/MIP3α secretion, impairing Langerhans cell migration (46), while HPV-positive cells secrete elevated levels of G-CSF, IL-6, IL-8, promoting the recruitment of tumor-associated neutrophils and M2 macrophages (47).

### 3.3 Impact of tumor-associated macrophages on cervical cancer prognosis

Increased TAM infiltration is associated with poorer patient prognosis (48–50). High-risk HPV infection is the primary cause of cervical cancer; however, immune responses against HPV antigens eliminate most infections and precursor lesions, with only a minority of infected individuals developing persistent infections leading to malignancy. Interleukin-10 (IL-10) inhibits the production of other cytokines, such as IL-2, IFN-γ, IL-12, and TNF-α, and is associated with downregulation of MHC class I molecules, resulting in reduced Th1 responses. In cervical cancer, TAMs secrete IL-10, inducing the proliferation of HPV-specific regulatory T cells and suppressing the anti-tumor activity of effector T cells, thereby contributing to adverse prognosis. During tumorigenesis and progression, cancer cells proliferate by absorbing nutrients via blood vessels, a process closely linked to TAMs (38). TAMs promote tumor angiogenesis and metastasis, suggesting that TAMs may facilitate the extensive growth of new blood vessels through factor secretion, synergistically promoting the malignant progression of cervical cancer (51).

**Figure 1:**
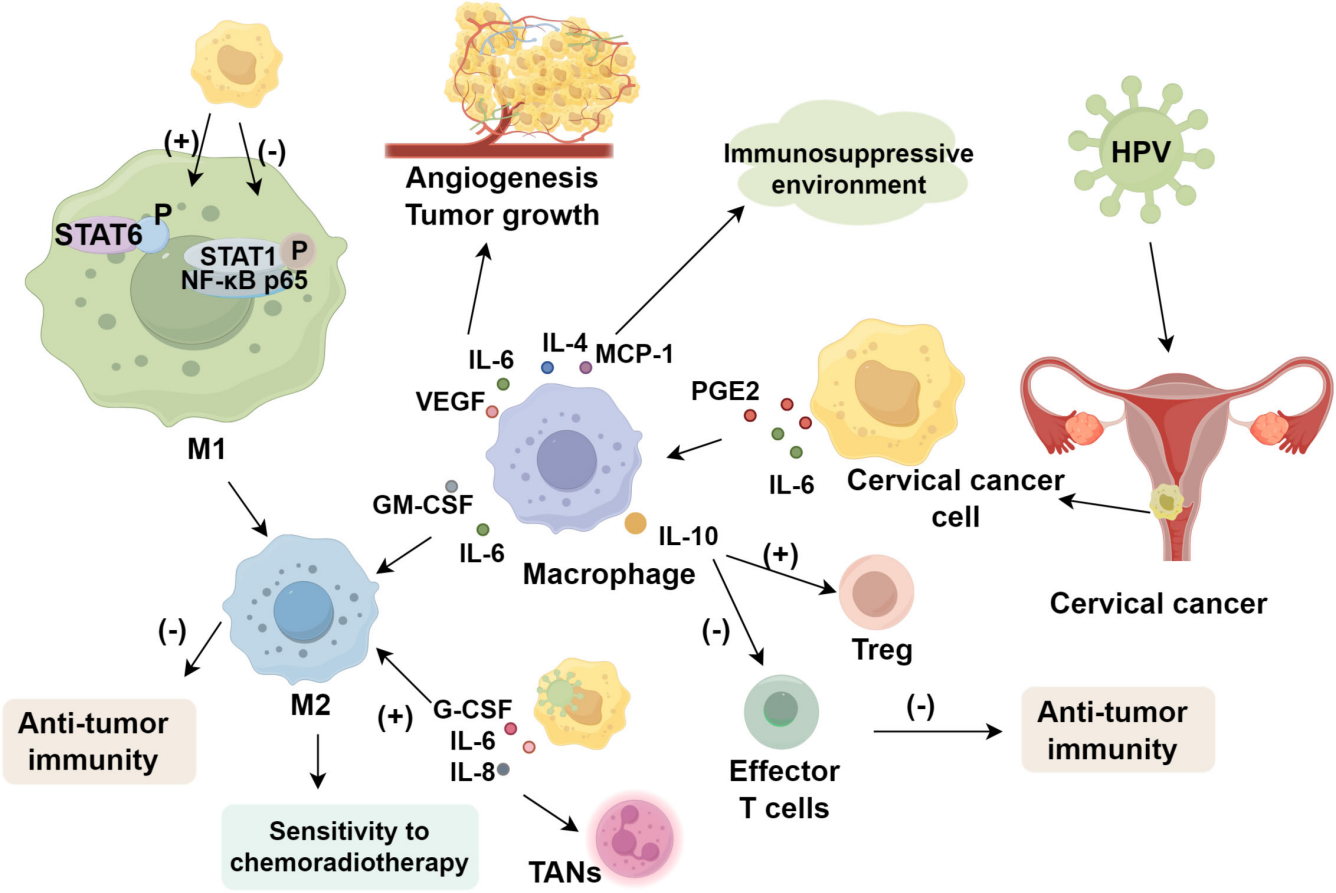
The Role of tumor-associated macrophages in cervical cancer.

TAMs are closely associated with recurrence and metastasis in cervical cancer. CD163 and CD68 are common TAM markers. The increased CD163^+^ macrophage count was significantly associated with reduced recurrence-free survival, whereas CD68^+^ macrophages were not correlated with recurrence in Stage I squamous cell carcinoma of the cervix (52). Moreover, M2-polarized TAMs reduce sensitivity to chemoradiotherapy, and a higher M1/M2 ratio independently predicts unfavorable survival. Targeting macrophage polarization to prevent M2 differentiation effectively impedes tumor progression (53, 54), thereby improving cervical cancer prognosis (**Figure 1**).

## 4 Therapeutic targeting of TAMs

### 4.1 Phenotypic remodeling of TAMs

M1-type macrophages show anti-tumor, pro-inflammatory roles in cervical cancer, whereas M2-type macrophages correlate with poor prognosis and are more prevalent in cervical cancer tissues than non-tumorous samples (55, 56). 6Because macrophages are highly plastic, promoting M1 polarization and inhibiting M2 differentiation effectively treats cervical cancer via phenotypic remodeling. Such a strategy fosters monocyte differentiation into M1, curbs M2 polarization, and represents a viable therapeutic intervention for cervical cancer.

#### 4.1.1 Vaccines

To date, three prophylactic HPV vaccines, like bivalent, quadrivalent, and nonavalent, have been approved for preventing common HPV infections (57). However, current prophylactic HPV vaccines cannot eliminate existing HPV infections or interfere with the progression of precancerous lesions to malignancy (58). CHE YX et al. (59) employed a therapeutic vaccine containing HPV16 E7 43-77 peptides and adjuvants to treat cervical cancer in murine models, observing a downregulation of IL-10 and TGF-β expression in M2-type macrophages and an upregulation of CXCL9 and CXCL10 chemokines in M1-type macrophages. This induced significant reduction in the percentage of M2-type TAMs, decreased tumor microvascular density, and reduced Ki-67 indices, suggesting a macrophage shift from M2 to M1. Similarly, Sonia et al. (60) demonstrated that HPV16 E7 peptides and CpG ODN reduced TAMs and genital tumor growth. Moreover, low-dose naltrexone decreases the number of M2-type macrophages and reduces serum IL-10 secretion, thereby inhibiting cervical cancer progression (61).

#### 4.1.2 TAM-derived exosomes

Exosomes are extracellular vesicles containing nucleic acids, proteins, metabolites, and other bioactive molecules (62, 63), mediating “crosstalk” between tumor and immune cells, including TAMs. They serve as valuable mediators for TAM-targeted immunotherapy. In cervical cancer, tumor-derived exosomes promote TAM polarization toward M1, reducing PD-1 expression (64). Exosome-derived miR-423-3p targets CDK4/p-STAT3, silencing IL-6 and inhibiting M2 polarization (65). Inflammatory factors widely involve in diseases’ progression (66–69). In DC/HPV16 E7, silencing CAT2 in exosomes induces M1 differentiation, increasing IL-12 and TNF-α and curtailing tumor growth (70). SEPT9 methylation confers radio-resistance via miR-375 “crosstalk” to TAMs, favoring M2 polarization and tumor progression (71). TAM-derived exosomes also affect ferroptosis by transferring miR-660-5p through the IL-4/IL-13/p-STAT6 pathway, suppressing ALOX15 (72). Due to their wide distribution, abundance, stability, and plasticity, exosome-based technologies hold significant promise for cervical cancer therapy. Hence, understanding their mechanisms is essential for developing novel therapeutic strategies and improving patient prognosis in cervical cancer. By harnessing these vesicles, clinicians may optimize treatment outcomes.

#### 4.1.3 Mixed lineage kinase domain-like protein

Necroptosis, a programmed cell death mechanism, triggers pro-inflammatory cytokines and anti-tumor immunity, thereby contributing to tumor necrosis. Hence, molecules in necroptosis pathways are promising therapeutic targets (73). MLKL, the key necroptotic effector, undergoes RIPK3-mediated phosphorylation, oligomerizes, relocates to the plasma membrane, and disrupts membrane integrity, a hallmark of necroptosis (74). Cervical cancer cells may limit M1 macrophage polarization by reducing macrophage necroptosis, particularly in HPV-positive contexts (75). Plasma MLKL and HPV DNA are readily measurable in clinical labs, suggesting their combined detection in cervical cancer diagnosis and disease monitoring. Additionally, blocking MLKL expression might regulate macrophage polarization, offering a novel therapeutic strategy for cervical cancer. Future studies are further needed to elucidate MLKL’s clinical role in both prevention and therapy.

### 4.2 Inhibition of TAM viability

TAMs drive cervical cancer proliferation, invasion, and metastasis, making their depletion a key therapeutic strategy. Metastasis is initiated by tumor cell dissemination and the acquisition of invasive capabilities, often mediated by epithelial-mesenchymal transition (EMT). The TME, comprising TAMs, extracellular matrix, hypoxia, and tumor-mesenchymal interactions, fosters EMT (76). Low-dose naltrexone (LDN) has been shown to reduce TAMs *in vitro*, suppress M2-type macrophage-mediated cervical cancer proliferation, invasion, and migration, promotes apoptosis, and inhibits EMT (61), positioning it as a potential adjunct therapy targeting TAM survival to curb tumor progression. Chlorophosphonates induce TAM apoptosis and inhibit tumor growth in HPV16 E6/E7-expressing TC-1 murine models without affecting TC-1 cell viability (77). Additionally, mitomycin C (MMC) combined with MG132 enhances FasL expression in TAMs both *in vivo* and *in vitro*, amplifying their bystander effect on cervical cancer cells and suppressing tumor growth.

Angiogenesis is a critical process in tumor progression. DiNardo et al. (78) reported that zoledronic acid (ZA) in K14-HPV16 transgenic female mice suppresses MMP9 in TAMs, reduces VEGF-receptor binding, and suppresses angiogenesis, thereby inhibiting cervical cancer cell proliferation and metastasis. Moreover, chlorophosphonate-loaded liposomes deplete TAMs, boost tumor-specific CD8^+^ T-cell responses, and inhibit TC-1 tumor growth in murine models (79).

### 4.4 Regulation of TAM recruitment to the microenvironment

#### 4.4.1 Interference with chemokines to inhibit M2 macrophage recruitment

Cytokines and chemokines within the TME drive monocyte and macrophage recruitment to tumors and their subsequent differentiation into the TAM phenotype. As previously discussed, administering a cervical cancer vaccine containing HPV16 E7 43-77 peptides to TC-1 murine models alters the immune microenvironment, decreases the expression of chemokines such as CCL2 and CCL5, suppresses myeloid-derived suppressor cell (MDSC) recruitment to tumors, and significantly reduces M2-TAMs within tumors (59). The natural anticancer compound swainsonine competitively inhibits α-mannosidase, modulates TAM phenotypes, and suppresses the secretion of CCL2 and IL-10, thereby reducing macrophage recruitment within the microenvironment (80). Under hypoxic conditions in cervical cancer, high expression of neuropilin-1 (Nrp-1) is significantly associated with TAM recruitment and migratory function. Researchers have found that interfering with Nrp-1 expression markedly impairs TAM migration (81).

#### 4.4.2 Nanomedicine delivery systems to enhance immune cell recruitment

Nanomedicine-based immunotherapy harnesses nanoparticles to deliver drugs or immune cells, enhancing efficacy and minimizing toxicity. Targeting TAMs in cervical cancer shows promise, exemplified by silica-based nanocomposites with miR-125a that shift macrophages to M1, reducing tumor growth (82). Nanoemulsions with TLR7/8 agonists increase M1/M2 ratios in bone marrow-derived macrophages, elevating MCP-1/CCL2 and MIP-1α/CCL3 to bolster immune cell recruitment (83). In HPV E6/E7-positive TC-1 tumor models, mannose-modified polyethyleneimine nanomicelles loaded with R-848 target dendritic cells and macrophages in draining lymph nodes, polarizing M2 to M1 macrophages, activating CD8^+^ T cells, and enhancing anti-tumor responses (84) (**Table 1**).

**Table 1:** Summary of TAM-targeted interventions in cervical cancer.

Targets	Agents	Mechanism
Phenotypic Remodeling	HPV16 E7 peptide-based vaccines; CpG ODN; Low-dose naltrexone (LDN)	Induce M1 differentiation; Inhibit M2 polarization; Shift cytokine milieu.
Exosome-Based Approaches	Exosomal miR-423-3p; DC/HPV16 E7 exosomes.	Deliver miRNAs or proteins to modulate macrophage polarization Inhibit IL-6 expression; Enhance M1 immune responses.
Necroptosis Induction	Targeting MLKL expression RIPK3/MLKL pathway modulation.	Trigger MLKL-mediated cell death; Enhance inflammatory signals to stimulate anti-tumor immunity.
Inhibition of TAM Survival	Chlorophosphonate-loaded liposomes; LDN; MMC+MG132 (FasL induction).	Deplete TAMs directly; Reduce pro-survival signals in TME. Promote apoptosis of M2 macrophages.
Anti-Angiogenic Approaches	Zoledronic acid (ZA); MMP9 inhibitors.	Suppress TAM production of VEGF/MMP9; Block tumor vascularization.
Inhibition of Recruitment	HPV16 E7 peptide vaccine; Swainsonine; Anti–Nrp-1 therapy.	Interfere with chemokine pathways (CCL2, CCL5); Block Nrp-1-mediated macrophage migration.
Nanomedicine Delivery	Silica-based nanocomposites (miR-125a); Nanoemulsions (TLR7/8 agonists); Mannose-modified PEI nanomicelles.	Utilize nanoparticles to deliver immunomodulatory agents; Enhance infiltration of immune cells; Polarize M2→M1 macrophages.

## 5 Conclusion

In conclusion, TAMs in cervical cancer predominantly exhibit an M2-like phenotype, contributing to tumor progression, immune suppression, and therapy resistance, while strategies targeting TAM reprogramming toward an M1 phenotype or reducing M2 polarization show promising therapeutic potential. Future research should focus on elucidating the molecular mechanisms of TAM plasticity, exploring combinational therapies such as vaccines and immune checkpoint inhibitors, and investigating HPV-mediated macrophage manipulation to develop novel immunomodulatory approaches. Clinically, TAM-targeted therapies could complement existing treatments, with biomarkers like CD163 and IL-10 guiding personalized interventions, ultimately improving tumor control and patient outcomes through optimized multimodal strategies.
